# Large Multicountry Outbreak of Invasive Listeriosis by a Listeria monocytogenes ST394 Clone Linked to Smoked Rainbow Trout, 2020 to 2021

**DOI:** 10.1128/spectrum.03520-22

**Published:** 2023-04-10

**Authors:** Sven Halbedel, Ida Sperle, Raskit Lachmann, Sylvia Kleta, Martin A. Fischer, Sabrina Wamp, Alexandra Holzer, Stefanie Lüth, Larissa Murr, Christin Freitag, Laura Espenhain, Roger Stephan, Ariane Pietzka, Susanne Schjørring, Guido Bloemberg, Mareike Wenning, Sascha Al Dahouk, Hendrik Wilking, Antje Flieger

**Affiliations:** a FG11–Division of Enteropathogenic bacteria and Legionella, Consultant Laboratory for Listeria, Robert Koch Institute, Wernigerode, Germany; b Institute for Medical Microbiology and Hospital Hygiene, Otto von Guericke University Magdeburg, Magdeburg, Germany; c FG35–Division for Gastrointestinal Infections, Zoonoses and Tropical Infections, Robert Koch Institute, Berlin, Germany; d Postgraduate Training for Applied Epidemiology (PAE), Robert Koch Institute, Berlin, Germany; e ECDC Fellowship Program, Field Epidemiology path (EPIET), European Centre for Disease Prevention and Control (ECDC), Solna, Sweden; f National Reference Laboratory for *Listeria monocytogenes*, German Federal Institute for Risk Assessment, Berlin, Germany; g State Institute for Food, Food Hygiene and Cosmetics, Bavarian Health and Food Safety Authority, Oberschleissheim, Germany; h Institute for Food of Animal Origin, Rhineland–Palatinate State Investigation Office, Koblenz, Germany; i Department of Infectious Disease Epidemiology and Prevention, Statens Serum Institut, Copenhagen, Denmark; j Institute for Food Safety and Hygiene, Vetsuisse Faculty, University of Zurich, Zurich, Switzerland; k Austrian Agency for Health and Food Safety, Graz, Austria; l Department of Bacteria, Parasites and Fungi, Statens Serum Institut, Copenhagen, Denmark; m Swiss National Center for Enteropathogenic Bacteria and Listeria, Institute for Food Safety and Hygiene, University of Zurich, Switzerland; Iowa State University

**Keywords:** cgMLST, ST394, Ny9, epidemiology, Europe, Ny

## Abstract

Whole-genome sequencing (WGS) has revolutionized surveillance of infectious diseases. Disease outbreaks can now be detected with high precision, and correct attribution of infection sources has been improved. Listeriosis, caused by the bacterium Listeria monocytogenes, is a foodborne disease with a high case fatality rate and a large proportion of outbreak-related cases. Timely recognition of listeriosis outbreaks and precise allocation of food sources are important to prevent further infections and to promote public health. We report the WGS-based identification of a large multinational listeriosis outbreak with 55 cases that affected Germany, Austria, Denmark, and Switzerland during 2020 and 2021. Clinical isolates formed a highly clonal cluster (called Ny9) based on core genome multilocus sequence typing (cgMLST). Routine and *ad hoc* investigations of food samples identified L. monocytogenes isolates from smoked rainbow trout filets from a Danish producer grouping with the Ny9 cluster. Patient interviews confirmed consumption of rainbow trout as the most likely infection source. The Ny9 cluster was caused by a MLST sequence type (ST) ST394 clone belonging to molecular serogroup IIa, forming a distinct clade within molecular serogroup IIa strains. Analysis of the Ny9 genome revealed *clpY*, *dgcB*, and *recQ* inactivating mutations, but phenotypic characterization of several virulence-associated traits of a representative Ny9 isolate showed that the outbreak strain had the same pathogenic potential as other serogroup IIa strains. Our report demonstrates that international food trade can cause multicountry outbreaks that necessitate cross-border outbreak collaboration. It also corroborates the relevance of ready-to-eat smoked fish products as causes for listeriosis.

**IMPORTANCE** Listeriosis is a severe infectious disease in humans and characterized by an exceptionally high case fatality rate. The disease is transmitted through consumption of food contaminated by the bacterium Listeria monocytogenes. Outbreaks of listeriosis often occur but can be recognized and stopped through implementation of whole-genome sequencing-based pathogen surveillance systems. We here describe the detection and management of a large listeriosis outbreak in Germany and three neighboring countries. This outbreak was caused by rainbow trout filet, which was contaminated by a L. monocytogenes clone belonging to sequence type ST394. This work further expands our knowledge on the genetic diversity and transmission routes of an important foodborne pathogen.

## INTRODUCTION

Listeriosis is a rare but severe foodborne infection with a high case fatality rate (1, 2). The disease is caused by the bacterium Listeria monocytogenes, which is widely present in the environment, from which it frequently enters the food production chain (3). Due to its ubiquitous occurrence, exposure to contaminated food and infections are quite common, but infections are usually kept under control by the human immune system (4, 5). However, listeriosis may develop into life-threatening invasive disease in elderly and immunodeficient persons, as well as pregnant women and newborns (6).

On average, the incidence of invasive listeriosis is low in Europe (0.4 cases/100,000 inhabitants); however, the incidences vary among the European countries (7), and a temporary increase of case numbers was reported over the last decade in several of them (8, 9). Case fatality is 13% for non-pregnancy-related invasive listeriosis in Germany according to notification data (9), but a mortality of >40% had been observed for patients with L. monocytogenes bacteremia 3 months after infection in a French study (2). In an international listeriosis survey, case fatality was estimated to reach 15% and 26% for pregnancy-associated and nonpregnancy cases, respectively (10). Such values are exceptionally high compared to other bacterial foodborne pathogens (1), and thus, L. monocytogenes is regarded as a major public health problem, imposing a high burden of disease on global society (10).

Control and hygiene measures during food production and storage may help to prevent contamination and pathogen growth in raw material and in processed food products, but despite such precautionary measures, large outbreaks of listeriosis do occur (11–15). Because of international food trade and the occurrence of persistent bacteria in food processing environments, such as in biofilms, listeriosis outbreaks often generate geographically widespread clusters involving more than one country (11, 16–19) and can be active over prolonged periods of time (20–22).

Germany implemented routine whole-genome sequencing (WGS) of clinical L. monocytogenes isolates (“molecular surveillance”) in 2018 to advance listeriosis surveillance and the recognition of listeriosis outbreak clusters (23). This revealed that approximately 70% of all listeriosis cases in Germany are part of such WGS clusters (23), among which the largest clusters included more than 100 patients (14). When epidemiological investigations generate hypotheses on possible vehicles, suspected food items can be analyzed for contamination by L. monocytogenes. WGS of such food-contaminating isolates and of food strains from routine investigations may then support the identification and subsequent elimination of the infection source through matching of WGS-based typing data from clinical and food samples (14, 24, 25). Early recognition of listeriosis clusters increases the chance that outbreaks are detected and stopped by public health interventions in real time (25, 26), which can reduce listeriosis incidence, as observed in Germany after introduction of WGS-based pathogen typing (9).

Several types of food items have caused large listeriosis outbreaks in Germany, but ready-to-eat meat products have been identified as the vehicles in the largest outbreaks (14, 20, 22) and also in the world’s largest listeriosis outbreak that affected South Africa in 2017 and 2018 (11). Salmon and other fish products are another known cause of listeriosis outbreaks (16, 27), and in a previous study, we estimated that 27% of listeriosis cases with suspected food vehicles in Germany are possibly caused by contaminated salmon products (19).

We here report the identification of a recent large cluster of invasive listeriosis cases, which was identified by molecular surveillance in Germany and caused by a rare ST394 clone. Further cases were recognized in neighboring countries after sharing of typing data. Epidemiological investigations and WGS of L. monocytogenes food isolates identified smoked rainbow trout filet from a Danish producer as the most likely vehicle. Our study illustrates the importance of international trade of food items in spreading foodborne outbreaks and the significance of fish products for understanding the epidemiology of listeriosis.

## RESULTS

### WGS detected a large listeriosis cluster in Germany, Austria, Denmark, and Switzerland.

**FIG 1:**
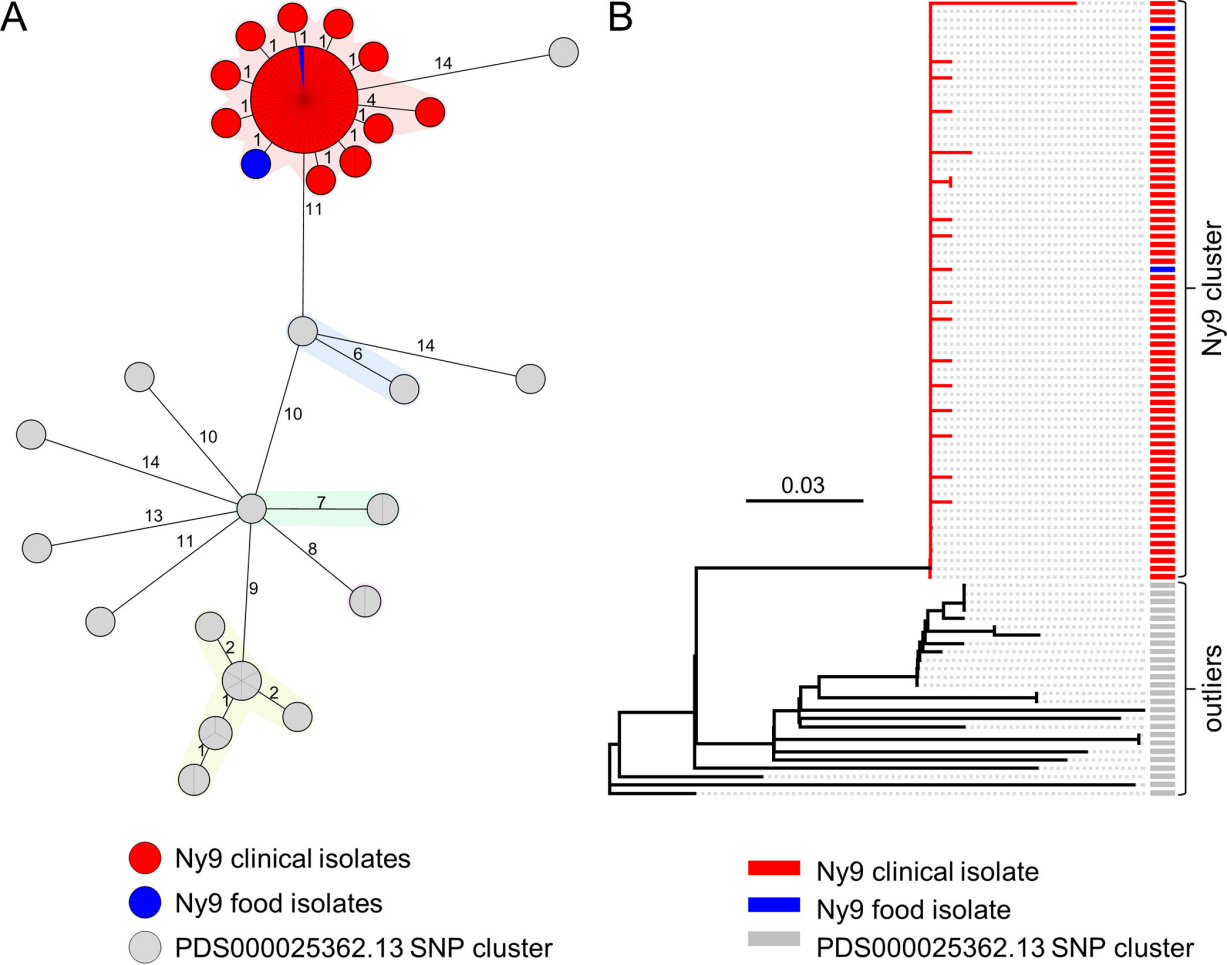
Identification of the Ny9 cluster by core genome multilocus sequence typing (cgMLST) and single nucleotide polymorphism (SNP) calling. (A) Minimum spanning tree of ST394 strains deduced from cgMLST data. Isolates are colored according to their origin, and clusters are highlighted by colored backgrounds. A pair of isolates was considered part of a cluster when they differed in ≤7 alleles. (B) Neighbor-joining tree for the same set of isolates calculated after read mapping to the closed genome of strain 20-05651 as the reference, which generated an alignment with a length of 201 SNPs after SNP filtering. Isolates are colored according to their origin. Scale is expressed as substitutions per site.

The German consultant laboratory (CL) for *Listeria* collects L. monocytogenes isolates from clinical infections in Germany and runs routine WGS for detection of disease clusters (23). This surveillance program covers approximately two-thirds of all notified cases of human listeriosis in Germany (9, 23). In November 2020, a sudden increase of isolates belonging to the core genome multi locus sequence typing (cgMLST) cluster types (CT) CT13516 and CT14488 was detected, and the new cluster was named Ny9. Isolates of this subtype belonged to molecular serogroup IIa, phylogenetic lineage II, seven-locus multi locus sequences typing (MLST) sequence type (ST) ST394, and clonal complex CC415; they had not been detected before in Germany and formed a remarkably clonal cgMLST cluster with 0 to 5 different pairwise alleles (median, 0; Fig. 1A). Information on the Ny9 cluster was shared with other European Union member states via the Epidemic Intelligence Information System (EPIS) communication platform of the European Center for Disease Prevention and Control (ECDC) on the 16 November 2020 (urgent inquiry UI-687). Raw sequencing data of a representative isolate (20-05651) was deposited on the European Nucleotide Archive (ENA) server under accession number SAMEA7540792. The EPIS notification returned recent closely related isolates in Austria (*n* = 2), Denmark (*n* = 2), and Switzerland (*n* = 1), indicating a possible cross-border distribution of the causative food item. Isolation of ST394 strains was occasionally reported in the literature (28–30), and several isolates were deposited at the National Center for Biotechnology Information (NCBI) pathogen detection portal, where they were designated PDS000025362.13 SNP cluster at the time of analysis (Fig. 1A and B; please note that this cluster has become part of the PDS000025362.26 SNP cluster at the time of revision). Deposited isolates were predominantly clinical isolates from the United Kingdom, and they formed a separate cluster and were isolated between 2013 and 2017. Also, several less related clinical and environmental isolates from the United Kingdom, the United States, and France (2012 to 2020) had been deposited. The ST394 isolates obtained from the NCBI database differed in 11 to 25 cgMLST alleles (median, 16) from the Ny9 cluster (Fig. 1A). In contrast, when three closed genomes of unrelated ST394 strains, which are currently available (accession numbers NC_021837, NZ_CP007199, and NZ_CP007200), were also considered in the analysis, overall diversity within ST394 isolates beyond the mere Ny9 and PDS000025362.13 clusters was considerably higher (0 to 104 cgMLST alleles; median, 4).

Weekly numbers of Ny9 isolates arriving at the CL peaked in the 45th calendar week in 2020 and subsequently declined. The last Ny9 isolate was collected on 31 January 2022. This last isolate differed in 4 or 5 alleles (median, 2) from all previous Ny9 isolates, which differed in 0 to 2 alleles (median, 0) only. Altogether, 68 Ny9 isolates were collected between September 2020 and January 2022 in the four affected countries (Germany, 63 isolates). A total of 52 of 68 Ny9 isolates were from blood, three were from synovial fluid, and one was from cerebrospinal fluid, confirming invasive disease, while the isolation source of 12 isolates remained unknown.

### Confirmation of the outbreak cluster through generation of a closed Ny9 genome, whole genome MLST (wgMLST), and single nucleotide polymorphism (SNP) calling.

The genome of one representative isolate from the central node of the cgMLST cluster (strain 20-05651) was reconstructed in a hybrid assembly approach using MinION and Illumina sequencing data. This yielded a single circular contig with a size of 2,920,067 bp and a GC content of 38% (Table 1), both of which were in the range that is characteristic for L. monocytogenes genomes (31). The 20-05651 genome was annotated using the NCBI Prokaryotic Genome Annotation Pipeline (PGAP), and its protein coding sequences were extracted to create a cluster-specific wgMLST scheme to further increase discriminatory power. Reanalysis of the Ny9 and PDS000025362.13 clusters with this Ny9-specific 2,842-locus typing scheme revealed 0 to 9 (median, 1) different alleles within the Ny9 cluster, whereas the allelic differences separating Ny9 from PDS000025362.13 isolates ranged between 26 and 63 (median, 54; Fig. S1). As a further approach to quantify the genetic relatedness of the Ny9 isolates, raw sequencing reads of all Ny9 and PDS000025362.13 isolates were mapped against the Ny9 reference genome of strain 20-05651 to allow for SNP calling. Isolates of the Ny9 cluster differed in 0 to 9 SNPs (median, 0), whereas the genetic distance of the PDS000025362.13 outgroup strains from the Ny9 cluster was 19 to 43 SNPs (median, 24; Fig. 1B), again indicating that they do not belong to the Ny9 cluster. Taken together, three different phylogenetic analyses consistently confirmed the existence of a highly clonal cluster of L. monocytogenes isolates, suggesting the presence of a common source for the underlying infection events.

**TABLE 1:** Key characteristics of the Ny9 outbreak reference strain 20-05651^*a*^

Isolate ID	20-05651
Outbreak cluster	Ny9
Source type	Clinical isolate
Source of isolation	Blood
Yr of isolation	2020
NCBI accession no.	CP093220.1
Sample accession no.	SAMEA7540792
Sequencing method	minION, Illumina
Long-read raw data	ERR8527660
Short-read raw data	ERR8527650
Genome size	2,920,067 bp
GC content	38%
CDSs	2,847
rRNA operons	6
tRNA genes	67
PAIs	LIPI-1
SSI-1/2	none
LGI1/2	none
Plasmids	none
Intact prophages^*b*^	One at tRNA^Arg^ (MNJ98_06240)
IS elements	5
Characteristic PSMCs	*clpY* (MNJ98_06730/*lmo1279*), *dgcB* (MNJ98_10045/*lmo1912*), *recQ* (MNJ98_14180/*lmo2757*)
PCR serogroup	IIa
Sequence type^*c*^	ST394
Clonal complex^*c*^	CC415
Complex type Ruppitsch scheme^*d*^	CT13516
Complex type Moura scheme^*e*^	CT8560

a CDS, coding sequence; cgMLST, core genome multilocus sequence typing; GC, guanine cytosine; IS, insertion sequence; LGI, Listeria genomic island; MLST, multilocus sequence typing; PAI, pathogenicity-associated island; PSMC, premature stop codon; SSI, stress survival islet.

b Obtained with PHASTER (43).

c Based on seven-locus MLSTs (67).

d Based on 1,701-locus cgMLSTs (56). cgMLST profiles and their alleles are available at https://www.cgmlst.org/ncs.

e Based on 1,748-locus cgMLSTs (28), available at https://bigsdb.pasteur.fr/listeria/.

### Epidemiological investigations and identification of the food source.

**FIG 2:**
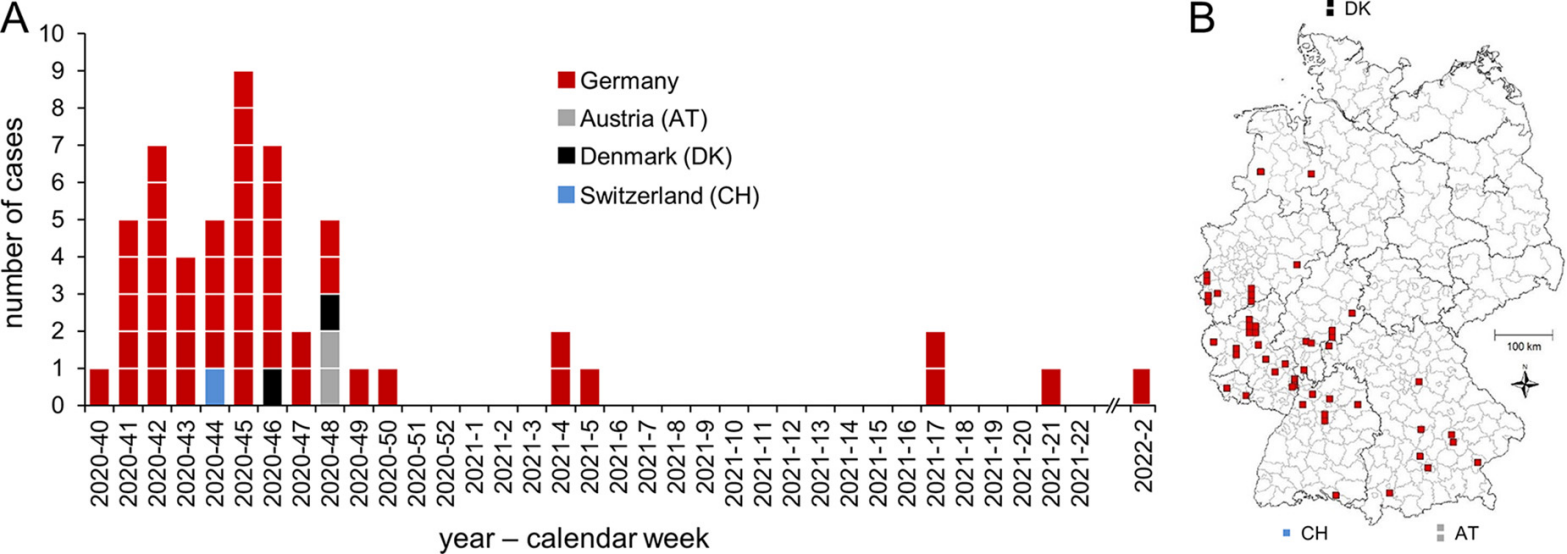
Chronology of outbreak progression and geographical distribution of cases. (A) Epidemic curve based on the notification week (sampling week for Denmark). The last Ny9 case from calendar week 2 of 2022, which was not considered during the epidemiological investigations, is shown separately. (B) Geographic distribution of the Ny9 outbreak cases between September 2020 and January 2022.

In total, 68 clinical isolates belonged to the Ny9 outbreak, of which all but the last temporally and genetically separated isolate were considered during epidemiological investigations. We identified 55 notified cases that could be allocated to these outbreak isolates. The cases were reported from October 2020 to January 2022 with the majority of cases notified from mid-October (week 41) to mid-November (week 46) in 2020. Seven cases were notified later in 2021, with the latest case notified in week 21 (Fig. 2A), and one case was reported much later in January 2022. Of the 55 cases, 50 were from Germany, 2 from Austria, 2 from Denmark, and 1 from Switzerland. In Germany, we identified cases in seven federal states primarily in the southwestern part of Germany (Fig. 2B). The states with the most cases were Bavaria (*n* = 8) and Rhineland-Palatinate (*n* = 10). Of all German patients, 22 (44%) were female, and their median age was 80 years (range, 0 to 94 years). Three cases were reported as deceased; for one case, another cause of death than listeriosis was notified, and for two cases, their cause of death was not reported. Two cases were pregnancy-associated. We interviewed 19 of the 55 cases about their food consumption prior to disease onset, and 16 (84%) recalled having eaten smoked trout (Germany, 13; Austria, 2; Denmark, 1; and Switzerland, 0). Smoked trout was by far the most frequently reported food item from the standardized questionnaire applied in Germany, with the second most frequently reported item being Gouda cheese (*n* = 11).

A Ny9 food isolate was detected inside an emptied and reclosed package of smoked rainbow trout filets from the waste bin of one of the cases in Rhineland-Palatinate (Germany). This food product was manufactured by Brand A in Denmark. On 2 December 2020, the Rapid Alert System for Food and Feed published a food warning due to the detection of L. monocytogenes in another batch of smoked rainbow trout filets of Brand A in an official sample that was taken on 19 October 2020 and exceeded the permitted limit for L. monocytogenes in ready-to-eat products (6.8 × 10^4^ CFU/g). At the time of notification, the use-by date of the affected batch (29 October 2020) had already expired. The genome sequence from this second food isolate was shared by the Bavarian Health and Food Safety Authority, and sequence comparisons confirmed its identity with the Ny9 cluster as well. Both food isolates differed in 0 to 5 cgMLST alleles (median, 1), 0 to 7 wgMLST alleles (median, 1), and in 0 to 8 SNPs (median, 1) from the clinical Ny9 isolates (Fig. 1A and B). The smoked trout from Brand A was sold in supermarkets in the four countries where Ny9 listeriosis cases were identified. Altogether, this strongly indicates that the Ny9 outbreak was caused by the consumption of rainbow trout filets of Brand A from the Danish producer. Investigations at the processing facility of Brand A in Denmark in December 2020 identified L. monocytogenes, but the identified isolate did not belong to the Ny9 cluster. In collaboration with the Danish Veterinary and Food Administration, the hygiene measures at the processing facility were intensified.

### Properties of the outbreak strain.

**FIG 3:**
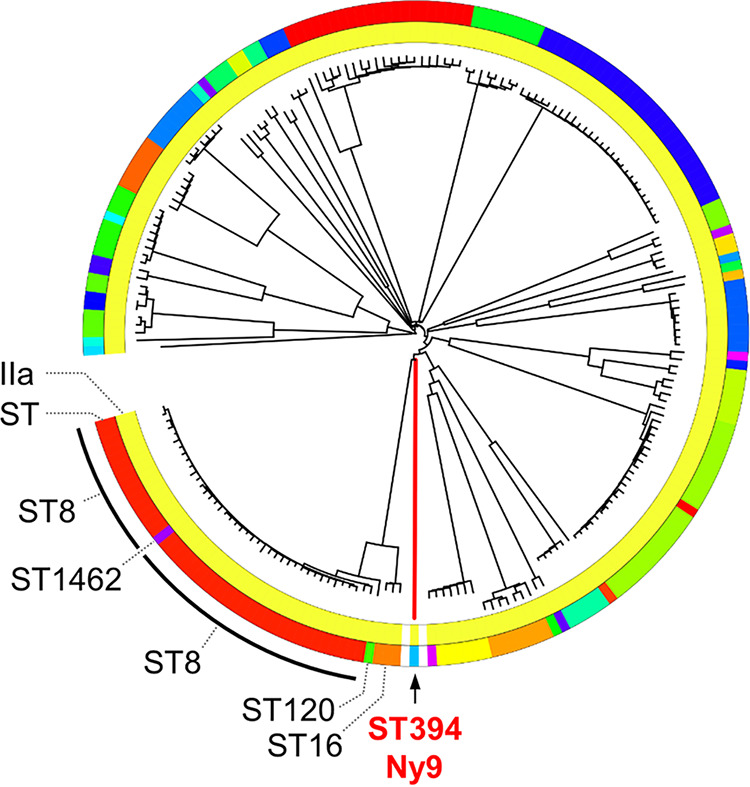
Phylogenetic classification of the Ny9 outbreak strain. Neighbor-joining tree based on core genome multi locus sequencing (cgMLST) data showing the position of the Ny9 (ST394) reference strain 20-05651 within a tree of representative serogroup IIa strains from a previously published nonredundant model population of clinical L. monocytogenes isolates from Germany (32). ST394 forms a deeply branching clade within the IIa strains and has ST8 (black arcs), ST1462 (purple), ST16 (orange), and ST120 (green) strains as next relatives. Of these, the ST8 strains are most closely related. The tree was calculated from data from 1,701-locus cgMLSTs and annotated with iTOL (65).

To define the phylogenetic position of the ST394 Ny9 cluster within the L. monocytogenes population, we compared ST394 strains with a previously published nonredundant model population covering the majority of clinical L. monocytogenes isolates from Germany (32) using cgMLST. The next relatives of the Ny9 strains found by this approach belong to ST8, from which the Ny9 reference strain 20-05651 is distinguished by 1,112 to 1,211 cgMLST alleles (median, 1,207), showing that both are phylogenetically very distant. Indeed, ST394 strains represent a deeply rooting phylogenetic branch within the serogroup IIa strains from this model population (Fig. 3). ST8 strains have repeatedly caused listeriosis outbreaks in Germany in the past, involving meat (20, 22) and salmon products as vehicles (19). To compare ST394 with an even wider phylogeny, we then used a collection of ~27,000 genomes previously downloaded from NCBI (representing the entirety of available L. monocytogenes genomes at the time of data download) (33) and extracted their cgMLST profiles. Comparison of the Ny9 ST394 cluster with the IIa isolates of this collection (~8,000 genomes) showed that the closest relatives of ST394 were ST361 (allelic distance, 372 to 396 cgMLST alleles), followed by ST918 (1,063 to 1,153 cgMLST alleles), ST376 (1,104 to 1,186 cgMLST alleles), ST398 (1,108 to 1,216 cgMLST alleles), ST199 (1,115 to 1,281 cgMLST alleles), ST16 (1,122 to 1,204 cgMLST alleles), and ST8 (1,129 to 1,215 cgMLST alleles). Among these, ST8 is the subgroup with the most isolates, and several closed ST8 genomes are also available.

**FIG 4:**
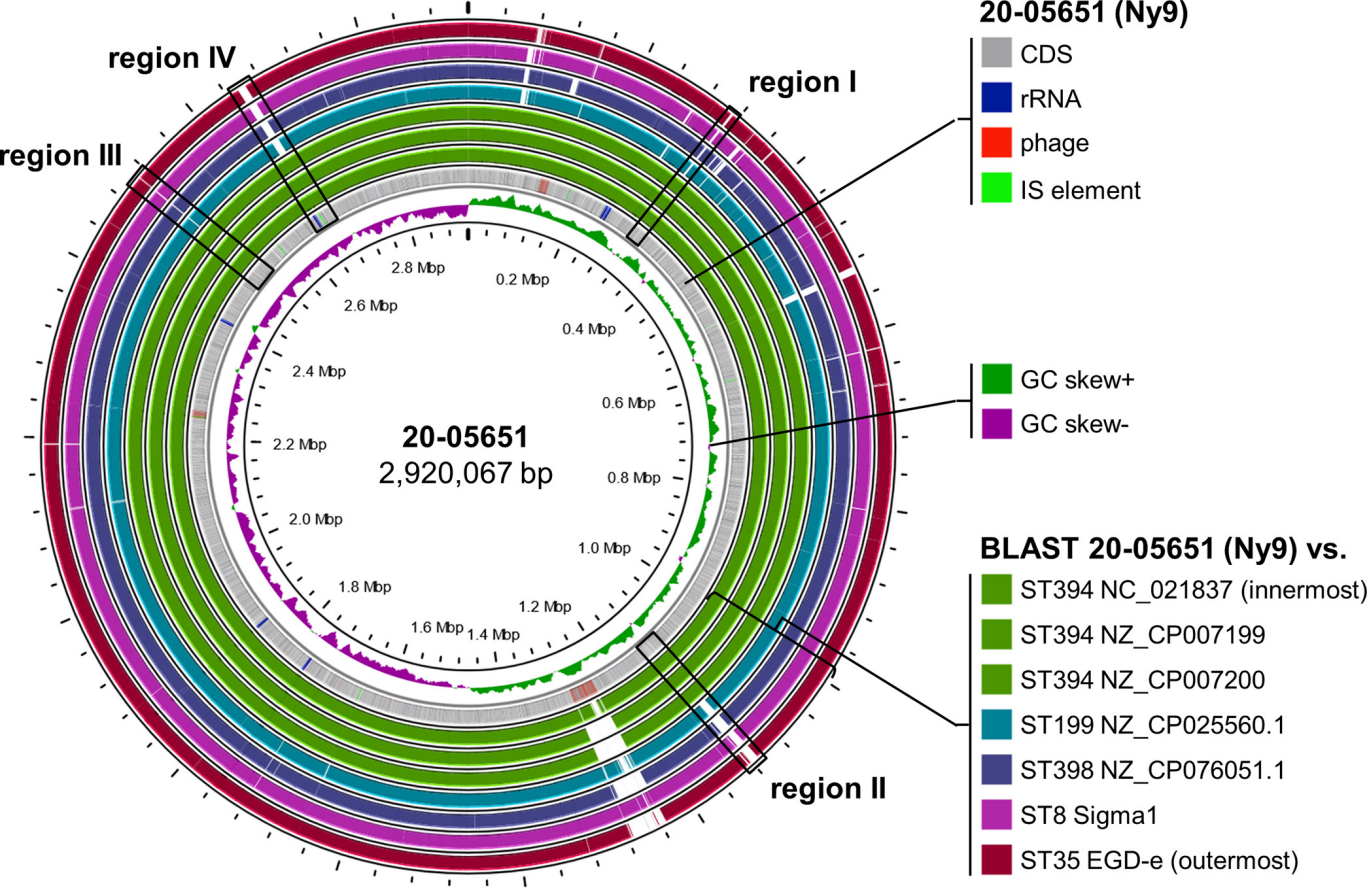
Comparison of the Ny9 genome with closed genomes of related subtypes. The genome map shows the Ny9 reference strain 20-05651 (gray circle). The positions of coding sequences (CDS), ribosomal RNAs (rRNAs), insertion sequences (IS), and putative phage regions are indicated. The presence/absence of Ny9 sequences in three available closed ST394 genomes, as well as in selected ST199, ST398, ST8 (Sigma1), and ST35 (EGD-e) genomes is illustrated by the several outer rings. ST394-specific regions I to IV are marked. The map was generated using Proksee (66). GC, guanine cytosine.

The Ny9 genome and the closed genomes of three other ST394 strains (strain J2-031, accession number NC_021837; strain 10-0933, accession number NZ_CP007199; and strain 10-0934, accession number NZ_CP007200) were compared to the closed genomes of selected ST199 (NZ_CP025560.1), ST398 (NZ_CP076051.1), and ST8 (CP064843) strains, as well as the EGD-e genome (ST35, NC_003210) as a reference. Closed genomes for the ST361, ST376, and ST918 strains, identified above as related to ST394, are currently not available. This identified 23 open reading frames in four chromosomal regions that were unique to ST394 (Fig. 4), and among them were five genes associated with an ATP-binding cassette (ABC) transporter locus (region I), a 12-gene cluster of unknown function (region II), a hypothetical gene (region III), and five CRISPR/Cas system associated genes (region IV) (Table 2; Fig. 4; Fig. S2). More detailed comparison of the Ny9 genome with the same set of reference genomes further revealed the presence of two Ny9-specific premature stop codons that truncated the *MNJ98_06730* (*clpY*/*hslU*/*lmo1279*) and *MNJ98_14180* (*recQ*/*lmo2757*) loci (Fig. S3). Moreover, the *MNJ98_10045* locus (*dgcB*/*lmo1912*) was also truncated in the Ny9 genomes (Fig. S3), but this truncation was also found in the ST394 isolates belonging to the PDS000025362.13 SNP cluster, even though it was absent in the three closed ST394 genomes (Fig. S3). RecQ is involved in the repair of DNA double-strand breaks (34, 35), but the Ny9 strain 20-05651 was as sensitive to ultraviolet (UV) light as the *recQ*^+^ Sigma1 strain (Fig. S4A). ClpY is the ATPase of the ClpYQ protease complex, implicated in control of biofilm formation in *Bacillus* species (36, 37); however, differences in surface attachment were not observed with the Ny9 strain (Fig. S4B). DgcB is a diguanylate cyclase and contributes to formation of cyclic-di-GMP (c-di-GMP) that stimulates exopolysaccharide production. A *dgcB* mutant phenotype was observed only when the phosphodiesterase genes for c-di-GMP hydrolysis were also deleted (38), and therefore, we did not pursue this further here.

**TABLE 2:** ST394 genes not found in selected ST199, ST398, and ST8 genomes or in strain EGD-e (as shown in Fig. 4)

Region	Locus tag	Annotation	Comment
I	MNJ98_01500	ABC transporter ATP-binding protein	ABC transporter locus
I	MNJ98_01505	ABC transporter	ABC transporter locus
I	MNJ98_01510	Hypothetical protein	ABC transporter locus
I	MNJ98_01515	Hypothetical protein	ABC transporter locus
I	MNJ98_01520	Hypothetical protein	ABC transporter locus
II	MNJ98_05540	Hypothetical protein	
II	MNJ98_05545	Hypothetical protein	
II	MNJ98_05550	Hypothetical protein	
II	MNJ98_05555	Cell surface protein	
II	MNJ98_05560	Hypothetical protein	
II	MNJ98_05565	Transcriptional regulator	
II	MNJ98_05570	Hypothetical protein	
II	MNJ98_05575	DUF87 domain-containing protein	
II	MNJ98_05580	Hypothetical protein	
II	MNJ98_05585	Replication protein	
II	MNJ98_05590	RNA helicase domain-containing protein	
II	MNJ98_05595	DUF3173 domain-containing protein	
III	MNJ98_12645	Hypothetical protein	
IV	MNJ98_13345	CRISPR-associated protein Csn2	CRISPR/Cas locus
IV	MNJ98_13350	CRISPR-associated endonuclease Cas2	CRISPR/Cas locus
IV	MNJ98_13355	CRISPR-associated endonuclease Cas1	CRISPR/Cas locus
IV	MNJ98_13360	CRISPR RNA-guided endonuclease Cas9	CRISPR/Cas locus
IV	MNJ98_13365	Hypothetical protein	CRISPR/Cas locus

The 20-05651 genome contained *Listeria* pathogenicity island (LIPI-1) as the only pathogenicity island and resistance genes other than those typically found in IIa strains or in EGD-e were not found. Likewise, stress survival islets SSI-1 and SSI-2 (39, 40) or the two *Listeria* genomic islands LGI1 and LGI2 associated with sanitizer and heavy metal resistance, respectively, (41, 42) were not present in the Ny9 genome. The insertion sequence (IS) finder (43) detected the presence of five insertion sequences (group IS3, family IS150) and PHASTER analysis (44) identified three putative phage regions (Fig. 4), of which two were either incomplete (13.2 kb at positions 2,237,969 to 2,251,253) or questionable (14.4 kb at positions 123,425 to 137,833; Fig. 4). However, one of the phage regions predicted by PHASTER was a complete 42.8-kb phage (1,234,197 to 1,277,032; Table 1) at the tRNA^Arg^ site with similarity to the *Siphoviridae* family phage B025 (45) and the phage found in strain LP-HM00113468 (NCBI accession number NC_049900.1).

**FIG 5:**
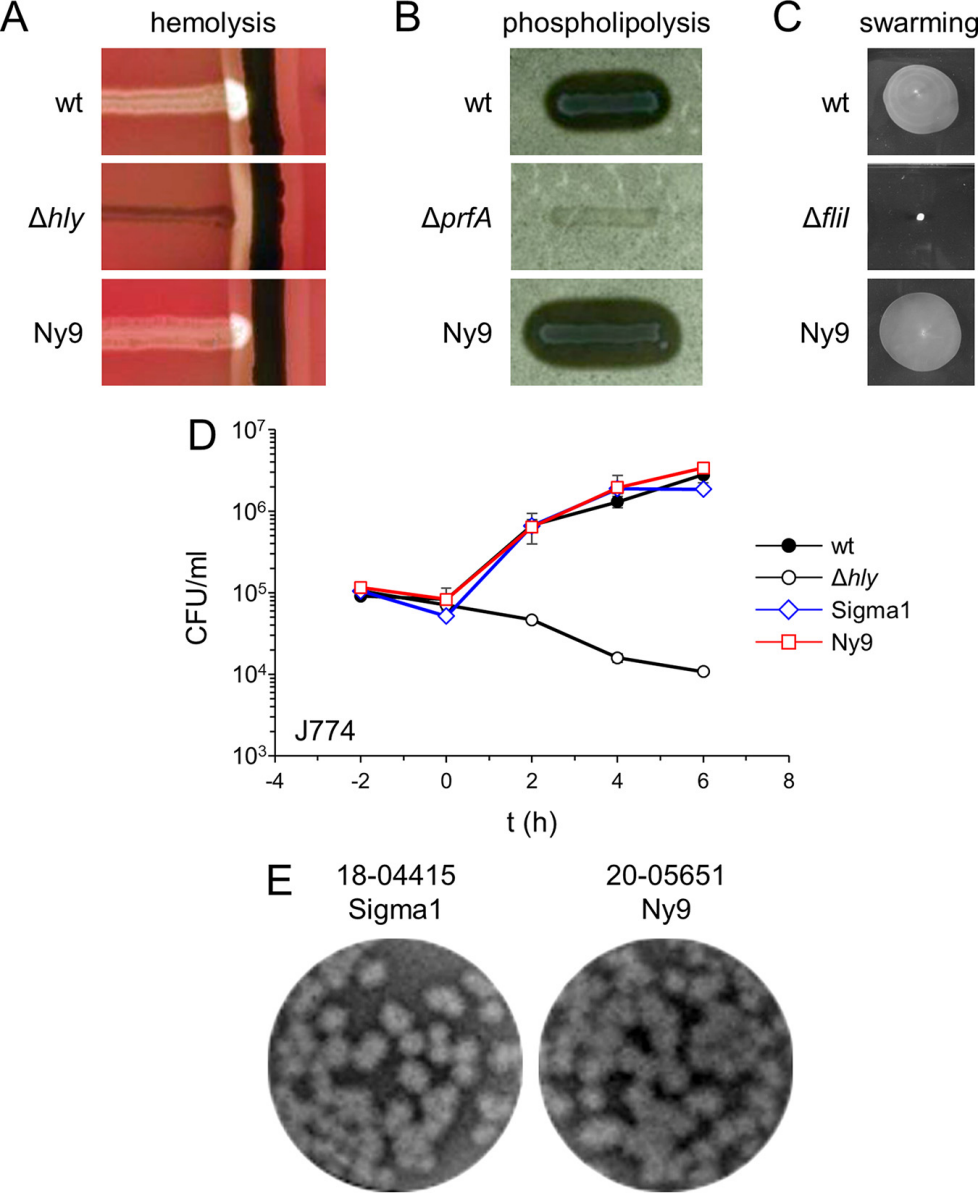
*In vitro* virulence profile of the Ny9 outbreak strain. (A) CAMP test showing background hemolysis and hemolytic synergism of L. monocytogenes strains EGD-e (wild type [wt]) and 20-5651 (Ny9) with S. aureus SG511. Strain LMS250 (Δ*hly*) was included as a control. (B) Phospholipolytic activity of L. monocytogenes strains EGD-e (wt) and 20-05651 (Ny9) on egg-yolk agar. L. monocytogenes strain Δ*prfA* was chosen as a control. (C) Motility of L. monocytogenes strains EGD-e (wt) and 20-05651 (Ny9) on soft agar. L. monocytogenes strain LMS3 (Δ*fliI*) was used as a control. (D) Intracellular growth of L. monocytogenes strain 20-05651 (Ny9) in J774 mouse macrophages. Strains EGD-e (wt), LMS250 (Δ*hly*), and 18-04415 (Sigma1 outbreak) are included for comparison. 18-04415 is one of the closest available ST8 relatives of strain 20-05651. The experiment was performed in triplicate, and average values and standard deviations are shown. The values labeled “-2 h” correspond to the inoculum. (E) Plaque formation assay to compare cell-to-cell spread of the Ny9 strain 20-05651 with strain 18-04415 (Sigma1 outbreak).

To detect possible alterations in virulence, several traits of strain 20-05651 were tested in various *in vitro* assays. However, strain 20-05651 showed a normal degree of hemolysis in the CAMP assay (Fig. 5A) and phospholipolytic activity similar to IIa strain EGD-e on egg-yolk agar plates (Fig. 5B). Likewise, flagellar motility of strain 20-05651 did not differ from EGD-e (Fig. 5C). Next, intracellular growth of strain 20-05651 in J774 macrophages was compared to EGD-e and the Sigma1 strain 18-04451 (ST8), but this did not reveal differences in growth rate or final bacterial loads between these three IIa strains (Fig. 5D). We also compared the ability of the Ny9 and Sigma1 strains to spread from cell to cell in a plaque formation assay, but there was no difference (Fig. 5E). Taken together, these results demonstrate that the clone causing the Ny9 outbreak, which belongs to a separated genomic clade within serotype IIa and is associated with an outbreak here, shows a virulence potential comparable to other IIa strains, despite the loss of *clpY*, *dgcB*, and *recQ* activities.

## DISCUSSION

This listeriosis outbreak with 55 cases (including three fatal cases) is the largest fish-product-related outbreak in Germany. Due to an underestimation of the incidence of L. monocytogenes septicemia and an under-reporting of cases and fatalities, as well as isolates for typing, the real disease burden of this outbreak is probably much higher. Microbiological and epidemiological lines of evidence consistently point to smoked trout as the most likely source, even though only one-third of the patients could be interviewed. Because of the small sample size, it was not possible to conduct an analytical epidemiological study, which would have provided stronger epidemiological evidence. Another limitation in the line of evidence is the fact that the outbreak clone was not found in the production facility; however, successful disinfection as an immediate hygiene measure in response to the product recall prior to targeted sampling during the investigation for the Ny9 outbreak source could be an explanation.

The Ny9 outbreak was caused by a ST394 clone. Genetic and phenotypic analysis demonstrated that this clone possessed a very similar virulence potential like the Sigma1 outbreak strain (22, 46), which was one of the closest relatives in our strain collection. ST394 strains have not been described so far in the context of large outbreak clusters. Interestingly, ST8 isolates, which were their closest, although distant, relatives, were under-represented in neurolisteriosis and maternal/neonatal infections and instead were associated with bacteremia (47). Concordantly, most of the Ny9 strains, for which the isolation source was known (93%), were isolated from blood, and only one was isolated from cerebrospinal fluid. This suggests that the virulence of ST394 and ST8 strains could be similar and determined by a specific gene set, but this needs to be further investigated, also using more sophisticated infection models.

The Ny9 outbreak presented here is one of the largest listeriosis outbreaks in Germany, where the largest outbreak, called Epsilon1a, affected 112 patients just 2 years earlier (14). Even though Ny9 and Epsilon1a were caused by different food items, there are several aspects that both outbreaks have in common: both outbreaks are characterized by a sudden and marked increase of case numbers and were of comparably short duration, and a high clonality of the outbreak isolates was observed. This differentiates the Ny9 and Epsilon1a outbreaks from other listeriosis outbreaks in Germany, which are normally active for years before they are detected and stopped and are characterized by lower degrees of clonality and, in some cases, even by the presence of more than one clone (19, 21, 22). Genetic diversity naturally increases with increasing outbreak duration due to progressing strain evolution (48), as long as the contaminating strain can replicate. Thus, high clonality is generally observed in outbreaks of the fulminant type or when the contaminant is given no chance for active replication at the site of contamination or inside the vehicle. Seen in that way, the epidemiological and genetic parameters of the Ny9 outbreak suggest a short-term contamination of the vehicle. Distribution of the contaminated batch in Germany and neighboring countries explains the geographic pattern of outbreak cases, which once more demonstrates the importance of cross-border collaboration in investigations of listeriosis outbreaks. While the majority of cases occurred in 2020, seven cases were reported in 2021 and 2022. This is also consistent with a short-term contamination; however, the source must have been active for some additional months on a lower level. Alternatively, some of the cases might potentially have consumed the trout filets later, after storing them in their freezers; however, these patients could not be interviewed.

The use-by date of the batch, in which the Ny9 clone was found, was end of October 2020, and at the same time, the presence of L. monocytogenes in the contaminated rainbow trout filets was detected by food safety officials. The disease cluster as such was recognized shortly thereafter, since the incubation period of listeriosis can vary between 3 and 70 days (49), and extra time is required for strain isolation, collection, and sequencing. Most probably, detection of the contamination and product recall had a great public health impact. Although most items of the contaminated batch(es) had most probably already been sold when the contamination was detected, consumers who noticed the product recall might have disposed of the contaminated trout. Furthermore, recognition of the Ny9 cluster by molecular surveillance could then have prevented further contamination of products from the same producer due to the initiation of targeted hygiene measures. This illustrates the importance of timely food sampling in official inspections and internal audits by food safety agencies and food producers, respectively, to prevent distribution of contaminated food items. In contrast, downstream molecular surveillance systems need to improve their turnaround times to increase their potential to influence outbreaks in real time, especially those of the fulminant type.

In addition to its impacts for public health, evolutionary genomics, and epidemiology of L. monocytogenes, our work also demonstrates that the *clpY*, *dgcB*, and *recQ* genes are not required for survival of L. monocytogenes or necessary to establish human listeriosis. ClpY is the poorly studied ATPase component of the ClpYQ proteasome (36, 50). Probably, loss of ClpY function can be compensated by other Clp ATPases. The *dgcB* gene encodes one of three diguanylate cyclases in L. monocytogenes for formation of the second messenger c-di-GMP (51, 52). Their redundancy might explain that one of them can be lost without impairing viability and virulence. Likewise, L. monocytogenes encodes a second *recQ*-like gene (*recS/lmo1942*), which could compensate the loss of the RecQ DNA helicase function, which is required for repair of DNA double-strand breaks (34).

## MATERIALS AND METHODS

### Bacterial strains and growth conditions.

Clinical L. monocytogenes strains were collected by the CL at the Robert Koch Institute (RKI) from diagnostic labs in Germany. The National Reference Laboratory for L. monocytogenes at the German Federal Institute for Risk Assessment (BfR) received food isolates that were isolated from local food authorities. Clinical isolates from Austria, Denmark, and Switzerland were collected by the Austrian Agency for Health and Food Safety (AGES, Vienna, Austria), the Staten Serums Institut (SSI, Copenhagen, Denmark), and the Swiss National Center for Enteropathogenic Bacteria and Listeria (Zurich, Switzerland), respectively. All clinical and food strains used in this study are listed in Table S1. L. monocytogenes mutant strains lacking the *hly* (33), *prfA* (53), *divIVA* (54), or *fliI* genes (54), all originating from EGD-e, as well as the L. monocytogenes Sigma1 outbreak strain 18-04415 (serogroup II, ST8, CT2521), which caused a nosocomial listeriosis outbreak in Germany in 2014 to 2019 that was associated with meat products (22, 46), were used as controls in different experiments. Escherichia coli TOP10 (Invitrogen, Waltham, MA, USA) was used as UV-sensitive indicator strain and E. coli K-12 C600 as a UV-resistant control (55). The strains were grown in brain heart infusion (BHI) broth (Oxoid, Wesel, Germany) or on BHI agar (Becton, Dickinson & Co., Franklin Lakes, NJ, USA) plates at 37°C.

### Genome sequencing and whole-genome sequence-based subtyping.

DNA was isolated by mechanical disruption using glass beads in a TissueLyser II bead mill (Qiagen, Hilden, Germany) or using the PureLink genomic DNA minikit (Invitrogen, Carlsbad, CA, USA) and quantified with Qubit double-stranded DNA BR or HS assay kits and Qubit fluorometers (Invitrogen) or using a NanoDrop spectrophotometer (Thermo Fisher Scientific, Waltham, MA, USA). The libraries were prepared using the Nextera XT DNA library prep kit or the NEBNext Ultra II DNA library prep kit (Illumina, San Diego, CA, USA) and sequenced on a MiSeq or NextSeq sequencer (Illumina) either in 1 × 150-bp single end or 2 × 250-bp or 2 × 300-bp paired end mode. Trimming of raw reads and contig assembly was performed in SeqSphere (Ridom, Münster, Germany) using Velvet as the assembler. SeqSphere was also used for extraction of *in silico* serogroups, seven-locus MLST STs, and 1,701-locus cgMLST CTs (56). Allelic cgMLST profiles were stored at https://www.cgmlst.org/ncs. Sequencing coverage ranged between 22- and 180-fold (median, 65-fold). cgMLST clusters were calculated in SeqSphere in the “pairwise ignore missing values” mode and defined as groups of isolates with ≤7 allele differences between neighboring isolates. Minimal spanning and neighbor-joining trees were calculated in SeqSphere.

For nanopore sequencing, DNA of isolate 20-05651 was prepared using a phenol-chloroform protocol as previously described (46). DNA was sequenced on a MinION (Oxford Nanopore, Oxford, UK) instrument using the SQK-RKB004 kit (Oxford Nanopore) in combination with a one-dimensional flow cell (Oxford nanopore) generating 347,050 long reads with 3,870 Gb. This data set was subsampled using filtlong (57) to 15,118 high quality long reads with a minimum length of 2 kb, summing up to 500 Mb.

### Generation of a closed Ny9 genome.

The genome of strain 20-05651 was reconstructed using Illumina and Oxford nanopore reads by a Unicycler (version 0.4.8) hybrid assembly (58). The assembly resulted in a circular genome with 2,920,067 bases and a coverage of 90.5-fold. The annotation was added by the NCBI PGAP (59).

### Single nucleotide polymorphism-based alignments and whole-genome MLST.

The batchMap pipeline (23) was used for mapping of sequencing reads against the reconstructed genome of strain 20-05651 as the reference, for generation of consensus sequences, and alignment generation. SNP filtering was performed with the SNPfilter pipeline (60) using an exclusion distance of 300 nt. SNP-based neighbor-joining trees were calculated in Geneious (Biomatters Ltd., Auckland, New Zealand). For wgMLST analysis, 2,842 nonredundant open reading frames of strain 20-05651 were extracted and used to create an *ad hoc* wgMLST scheme in SeqSphere. The same assembly and analysis procedures as for cgMLST were used.

### Epidemiological investigation into the source of the outbreak.

We defined outbreak cases as individuals notified with laboratory-confirmed listeriosis and identification of the cgMLST (56) complex types CT13516 or CT14488 with disease onset from September 2020. Demographic and disease relevant information on cases in Germany was collected from the national electronic surveillance system (Survnet). We conducted phone interviews with all cases in Germany who provided informed consent using a standardized exploratory questionnaire to assess food consumption at least 2 weeks prior to symptom onset. The results from the interviews were documented in an Excel spreadsheet and analyzed descriptively to identify the most commonly reported consumed food item among cases. Other categorical variables collected from Survnet were reported as proportions, range, and median.

### Phenotypic characterization.

Hemolysis was assayed in a CAMP test (61). For this purpose, Staphylococcus aureus strain SG511 was streaked on Mueller-Hinton agar plates containing 5% sheep blood right-angled to the L. monocytogenes strains to be tested. Hemolysis and hemolytic synergisms became apparent after overnight incubation at 37°C. An egg-yolk plate assay was used to compare phospholipolytic activity of L. monocytogenes strains (62). To this end, one egg yolk (15 mL) was resuspended in 15 mL sterile phosphate-buffered saline (PBS) buffer. This suspension was mixed with 200 mL molten LB agar containing 0.2% (wt/vol) activated charcoal and then poured as agar plate. L. monocytogenes strains were cultured on these plates, and zones of phospholipolysis became visible after 2 days of incubation at 37°C. Flagellar motility was tested on LB soft agar plates containing 0.3% agar. Strains were stab-inoculated, and the plates were incubated for 24 h at 30°C. To determine the resistance of bacterial strains to UV light, bacteria were grown overnight in BHI broth at 37°C. Optical densities were then adjusted to 2.0 (λ = 600 nm), and a 10-fold dilution series was prepared. A total of 5 μL of each dilution was spotted on two BHI agar plates, of which one was subjected to UV treatment afterwards (λ = 302 nm, 120 mJ, 2 s). Agar plates were incubated overnight at 37°C. Biofilm formation was analyzed in a microtiter plate format determining bacterial attachment to a plastic surface. The assay was performed as described previously (54).

### Infection experiments.

Assays to determine intracellular growth of L. monocytogenes strains in J774 mouse macrophages (ATCC TIB-67) and cell-to-cell spread in 3T3 mouse embryo fibroblasts were carried out as described previously (63, 64).

### Data availability.

Genome sequences were deposited at the European Nucleotide Archive (ENA) (https://www.ebi.ac.uk/ena) or at the National Center for Biotechnology Information (https://www.ncbi.nlm.nih.gov/). Accession numbers are listed in Table 1 and Table S1.
