# Supplementary material for: Large Multicountry Outbreak of Invasive Listeriosis by a Listeria monocytogenes ST394 Clone Linked to Smoked Rainbow Trout, 2020 to 2021
Source: Microbiol Spectr. 2023 Apr 10;11(3):e03520-22. doi: 10.1128/spectrum.03520-22 (PMC10269727; doi:10.1128/spectrum.03520-22)
Supplement: Supplemental file 2 — Figures S1-S4. Download spectrum.03520-22-s0002.pdf, PDF file, 0.4 MB [file spectrum.03520-22-s0002.pdf]

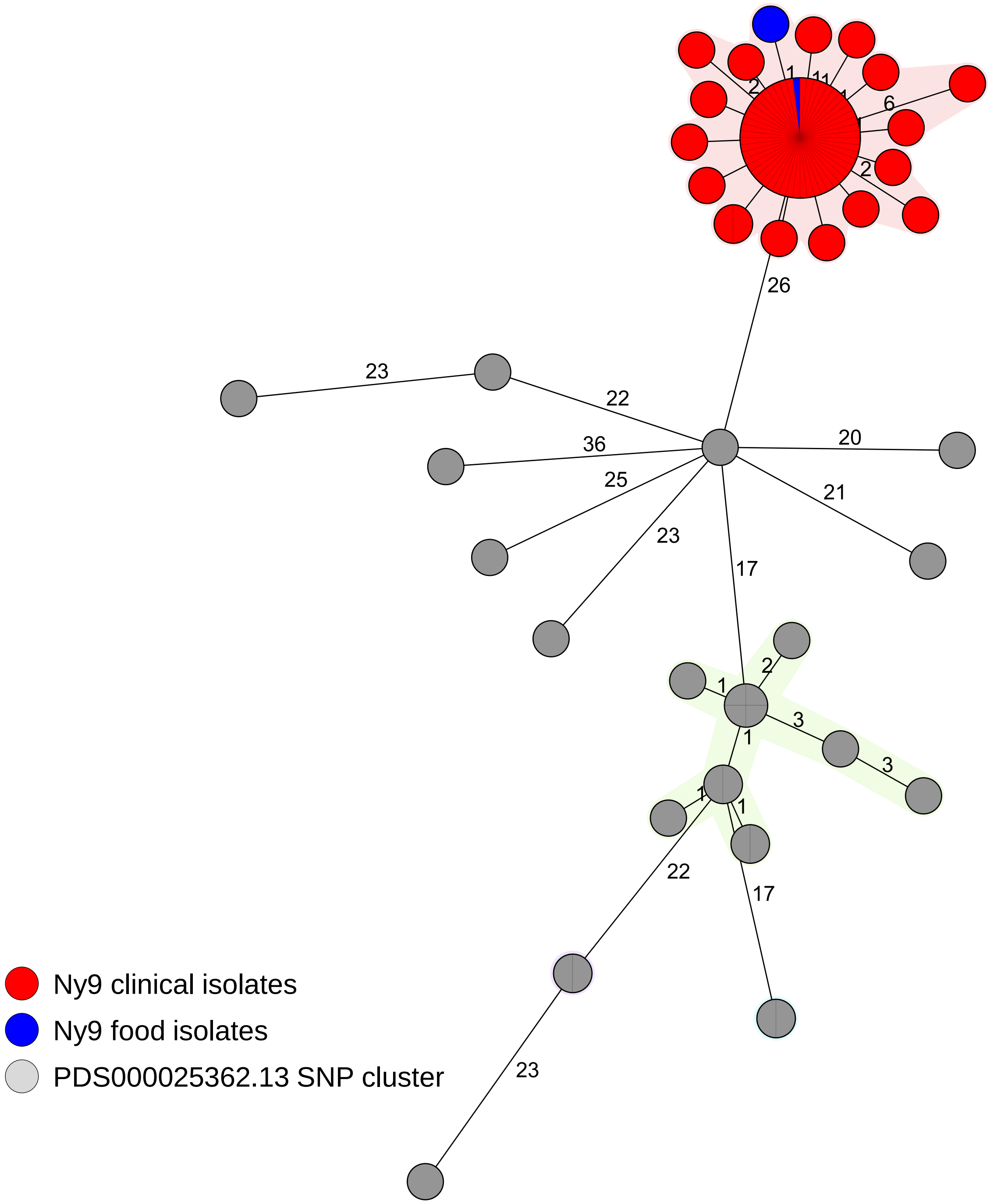

Fig. S1: Confirmation of the Ny9 cluster by whole genome MLST.
Minimum spanning tree calculated using a Ny9-specific wgMLST scheme with the 2842 loci of strain 20-05651 as targets with the same set of strains as in Fig. 1. Isolates are colored according to their origin and clusters are highlighted by colored backgrounds. A pair of isolates was considered as part of a cluster when they differed in ≤7 alleles.

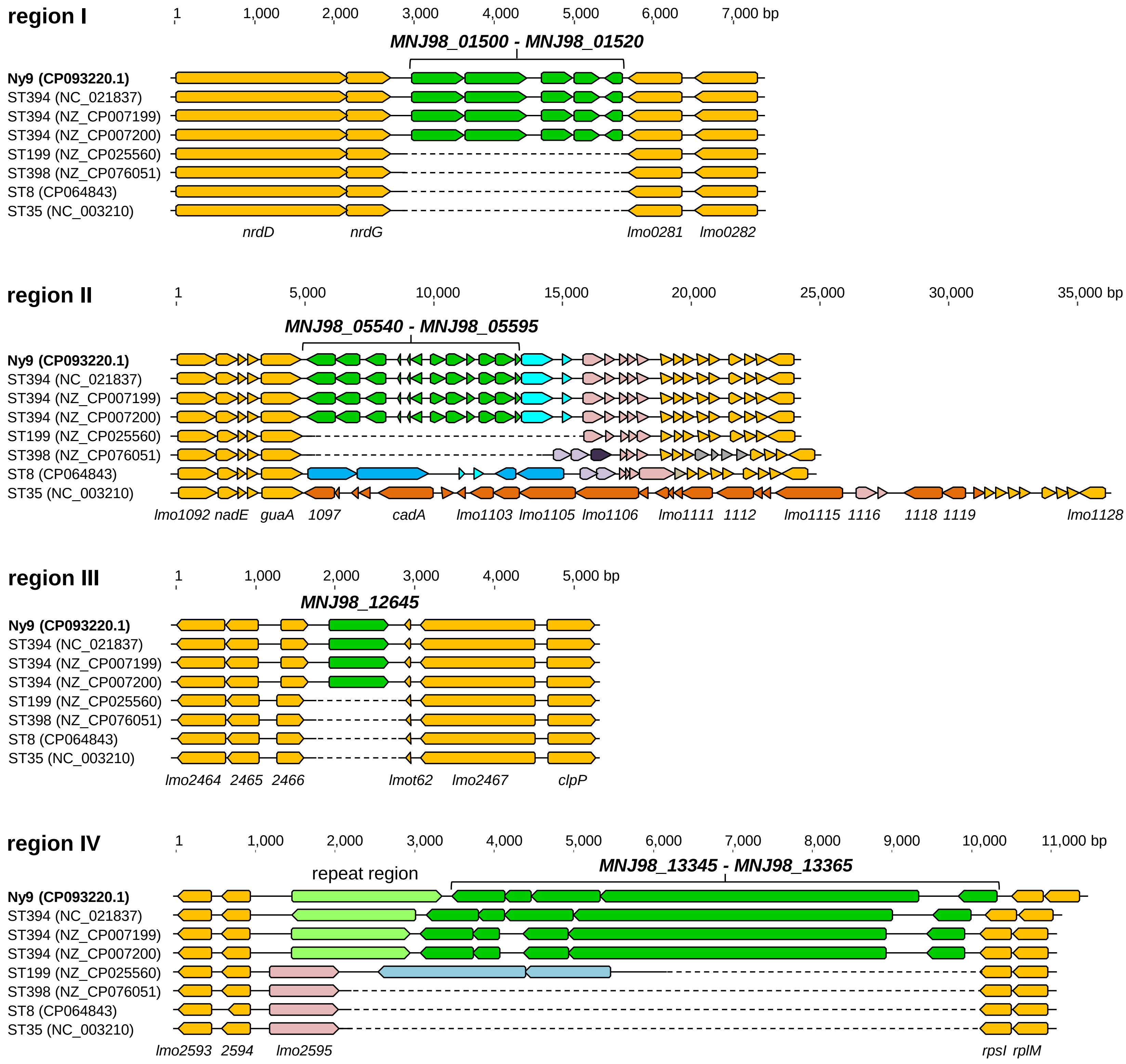

Fig. S2: Genes specific to ST394 strains.

Zoomed-in view of the four ST394-specific genomic regions identified in the comparison shown in Fig. 4. Genes equally present in ST394, ST199, ST398, ST8 (Sigma1) and ST35 (EGD-e) genomes are shown in orange. Genes unique to Ny9 and the three other ST394 genomes are shown in green, all other genes are shown in various colors. Genome sequence accession numbers are indicated on the left side.

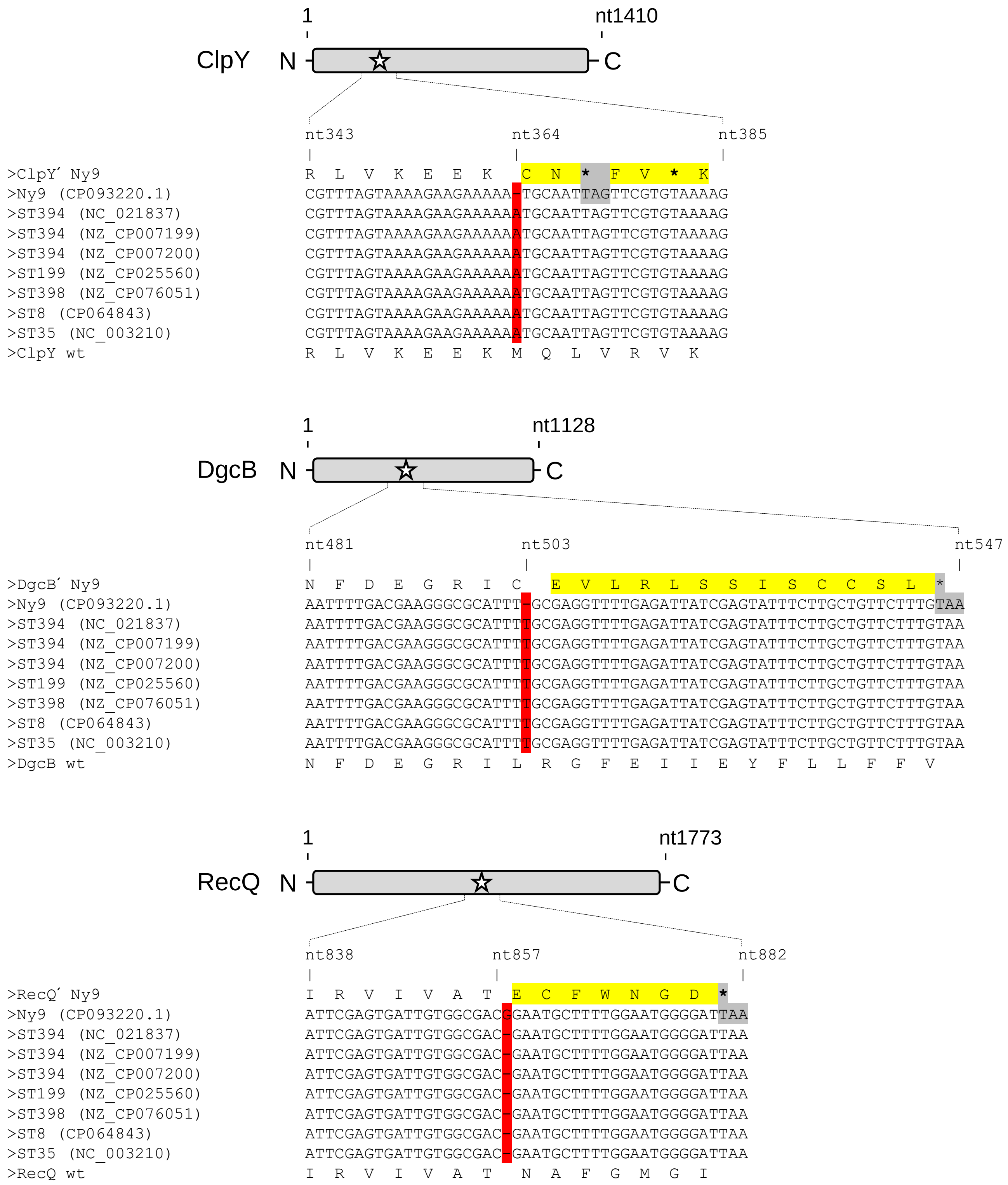

Fig. S3: Frame shift mutations inactivate the *clpY*, *dgcB* and *recQ* genes in the Ny9 clone.
Multiple alignments showing relevant sections of the *MNJ98_06730* (*clpY*/*hslU*/*lmo1279*), *MNJ98_10045* (*dgcB*/*lmo1912*) and *MNJ98_14180* (*recQ*/*lmo2757*) loci of the Ny9 reference strain 20-05651 (CP093220.1) and the homologous genes of strains J2-031 (ST394; NC_021837), 10-0933 (ST394; NZ_CP007199), 10-0934 (ST394, NZ_CP007200), PIR00545 (ST199, NZ_CP025560.1), 3BS90 (ST398, NZ_CP076051.1) and 18-04415 (ST8, CP064843) as well as EGD-e (ST35, NC_003210). Insertions/deletions of base pairs (red), frameshifted proteins sequences (yellow) and premature stop codons (grey) are highlighted by different background colors. Stars mark the position of insertions/deletions in relation to the full length gene.

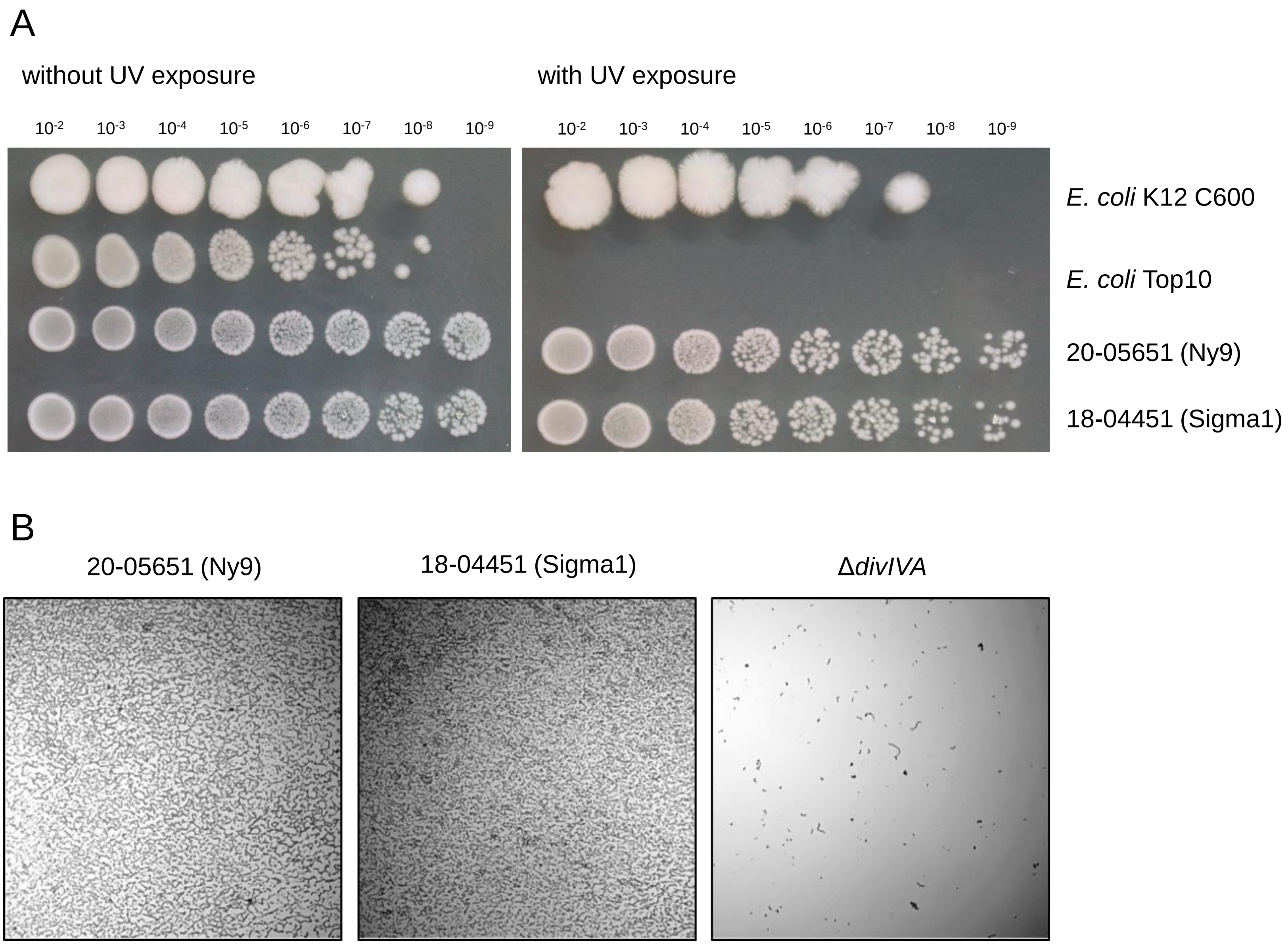

Fig. S4: The Ny9 clone shows normal UV sensitivity and biofilm formation.
(A) Spot dilution assay showing a dilution series of the Ny9 (20-05651, *recQ*$^{-}$) and the Sigma1 (18-04451, *recQ*$^{+}$) strains before and after exposure to UV light. *E. coli* K12 C600 (*recA*$^{+}$, UV-resistant) and *E. coli* Top10 (*recA*$^{-}$, UV-sensitive) were included as controls.
(B) Adherence of the Ny9 (20-05651, *clpY*$^{-}$, *dgcB*$^{-}$) and the Sigma1 (18-04451, *clpY*$^{+}$, *dgcB*$^{+}$) strains to the plastic surface of a microtitre plate. Non-adherent strain LMS2 (Δ*divIVA*) was used as a negative control.
